# Supplementary material for: Spatio-temporal propagation of cascading overload failures in spatially embedded networks
Source: Nat Commun. 2016 Jan 12;7:10094. doi: 10.1038/ncomms10094 (PMC4729926; doi:10.1038/ncomms10094)
Supplement: Supplementary Information — Supplementary Figures 1-19, Supplementary Notes 1-6, Supplementary Methods and Supplementary References [file ncomms10094-s1.pdf]

Supplementary Figures

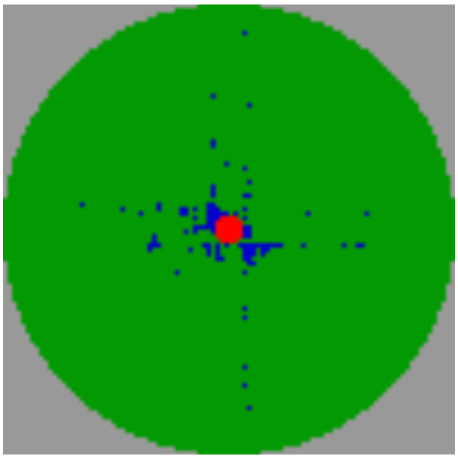

(a) $t = 1$

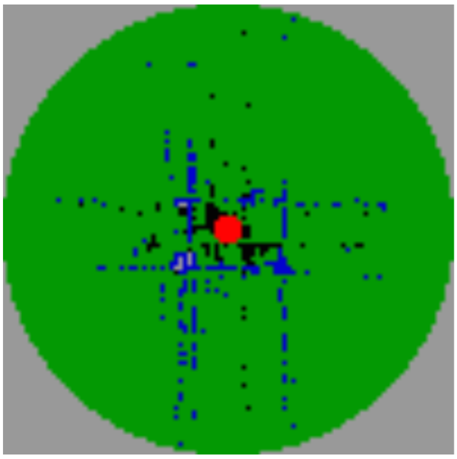

(b) $t = 2$

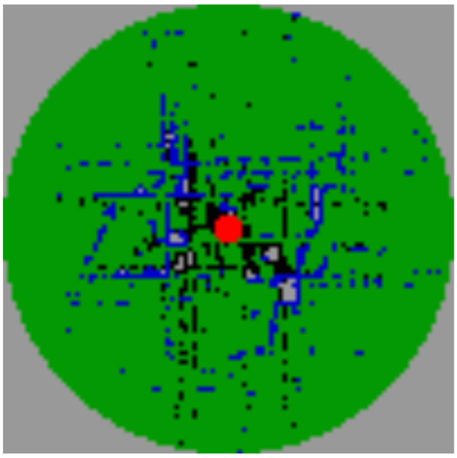

(c) $t = 3$

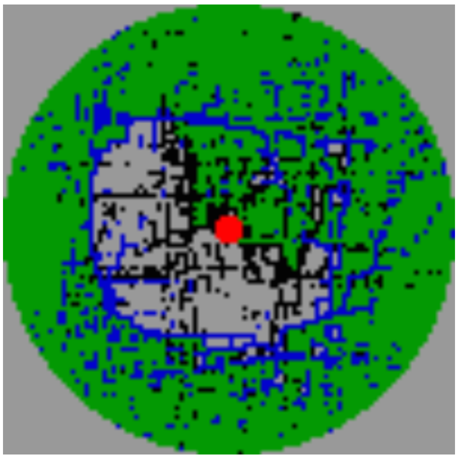

(d) $t = 4$

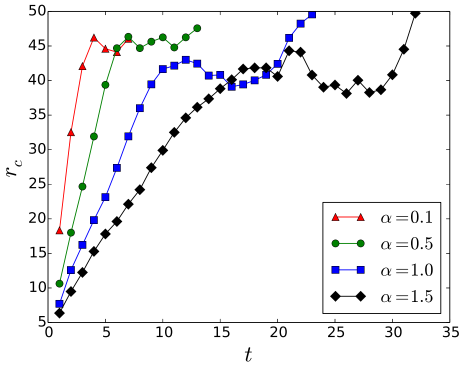

(e) radius of overloads

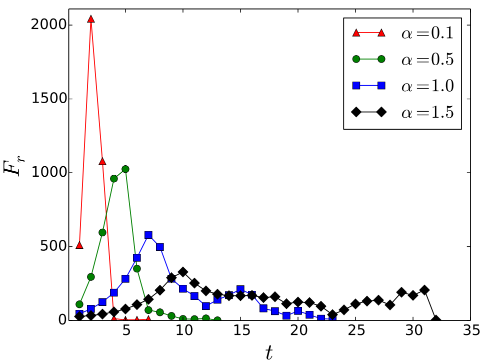

(f) number of failures

Supplementary Figure 1: **Overloads propagation in the weighted circular lattice.** (a) to (d) are the first four-steps of failure propagation. Here $L = 100$, $l = 6$, $\sigma = 0.1$ and the tolerance is set to $\alpha = 0.5$. Behaviors of $r_c(t)$ and $F_r(t)$ are shown in (e) and (f).

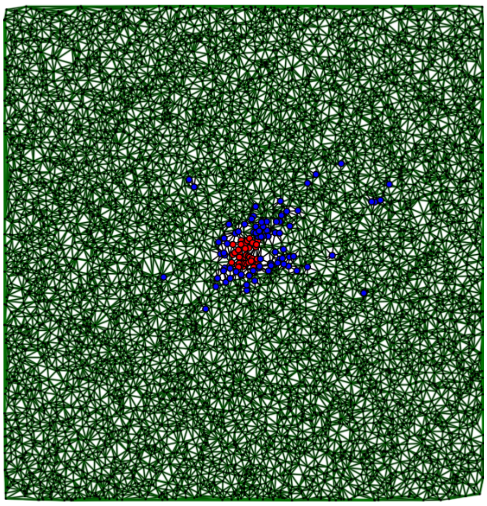

(a) $t = 1$

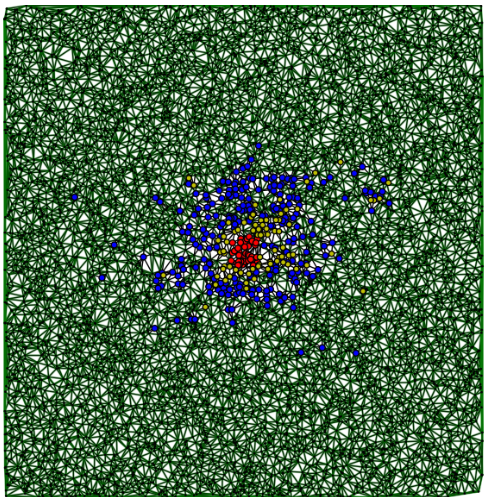

(b) $t = 2$

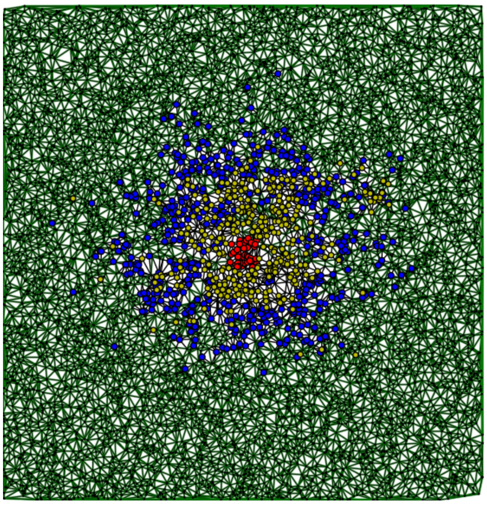

(c) $t = 3$

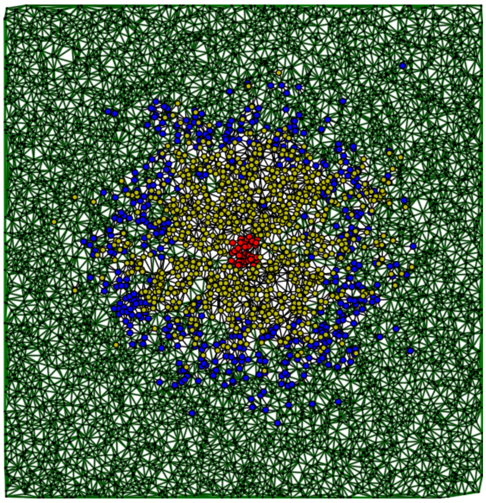

(d) $t = 4$

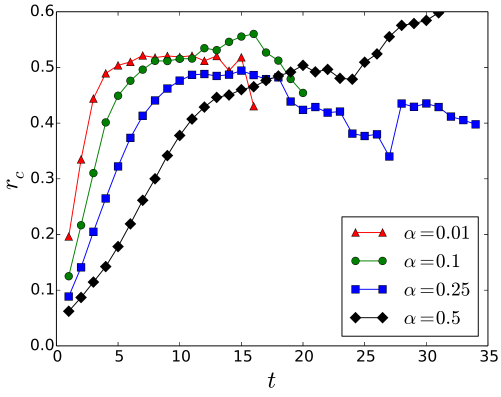

(e) radius of overloads

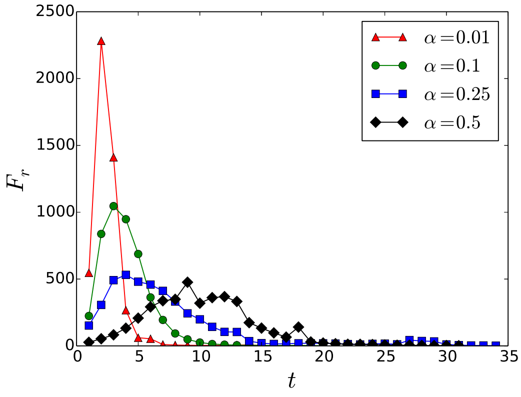

(f) number of failures

Supplementary Figure 2: **Overloads propagation in the weighted planar graph.** (a) to (d) are the first four-steps of failure propagation. Here the network size is 10000, we set $l = 0.06$, $\sigma = 0.1$ and the tolerance is set to $\alpha = 0.25$. Behaviors of $r_c(t)$ and $F_r(t)$ are shown in (e) and (f). Note that here the system linear size is $L = 1.0$.

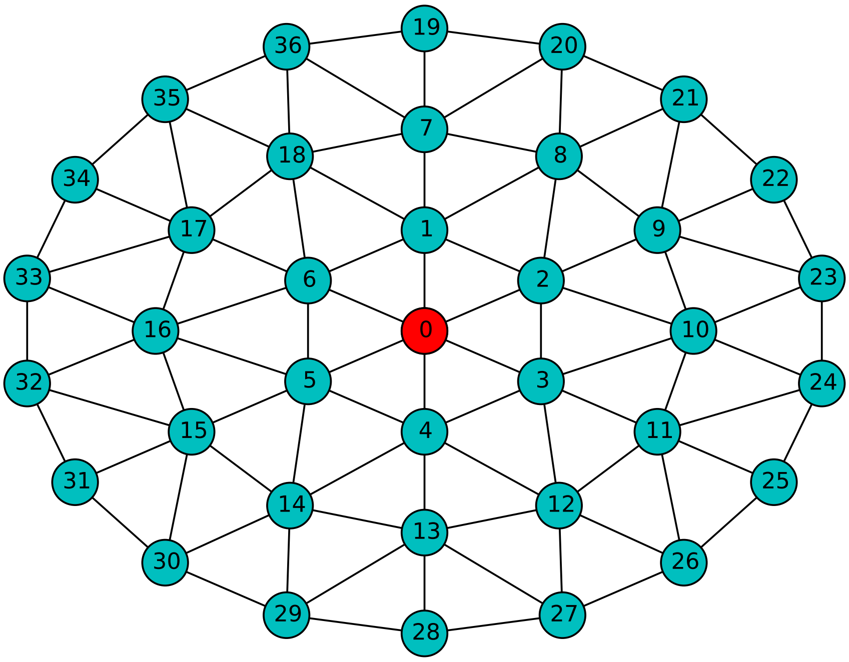

Supplementary Figure 3: **Example of circular plate network with 3 layers.**

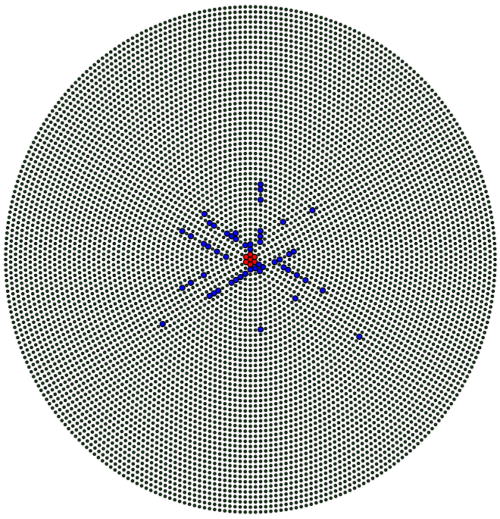

(a) $t = 1$

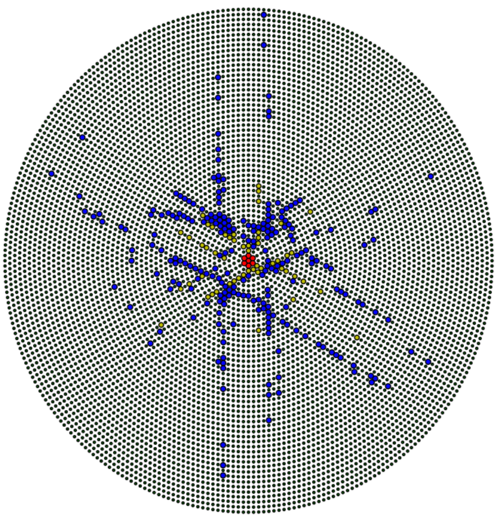

(b) $t = 2$

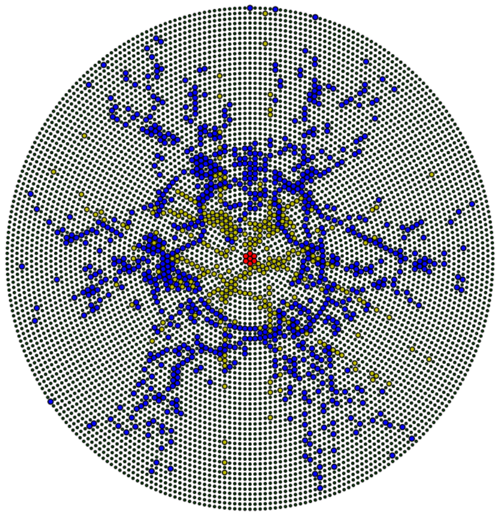

(c) $t = 3$

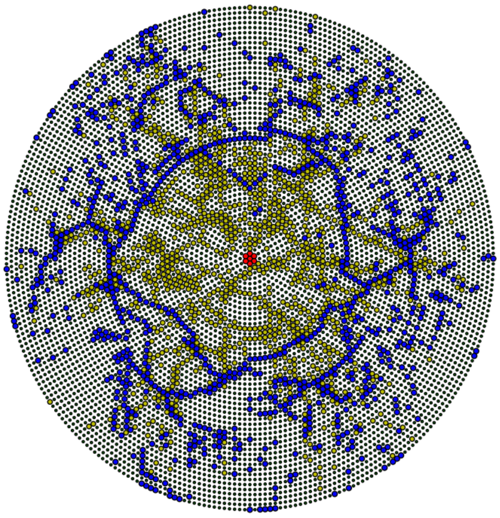

(d) $t = 4$

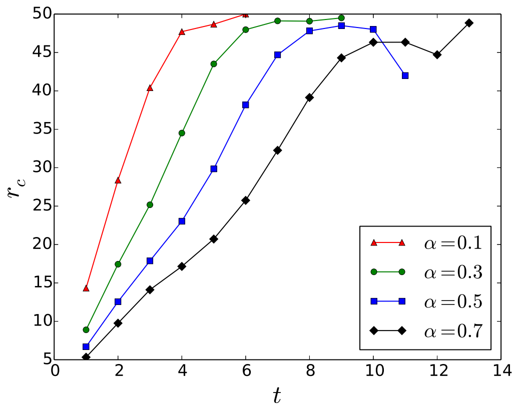

(e) radius of overloads

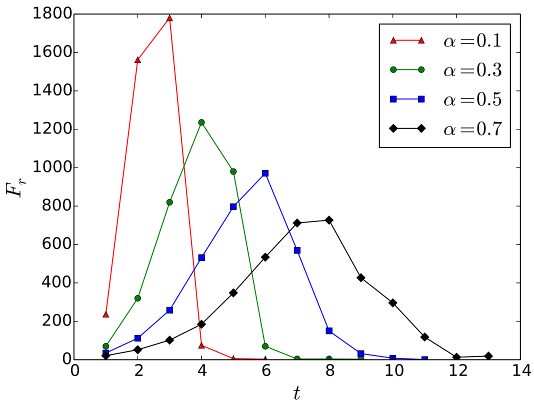

(f) number of failures

Supplementary Figure 4: **Overloads propagation in the weighted circular plate.** (a) to (d) are the first four-steps of failure propagation. Here the diameter of system is set to $L = 100$ and the tolerance is $\alpha = 0.3$. Behaviors of $r_c(t)$ and $F_r(t)$ are shown in (e) and (f).

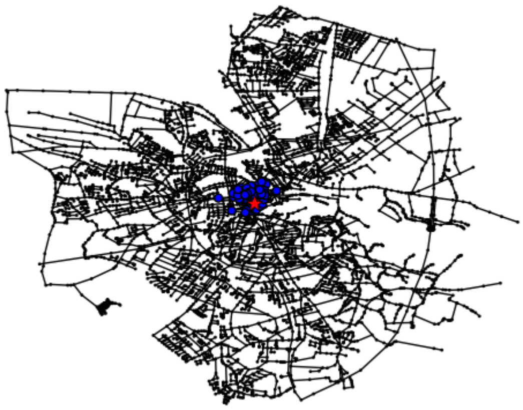

(a) $t = 1$

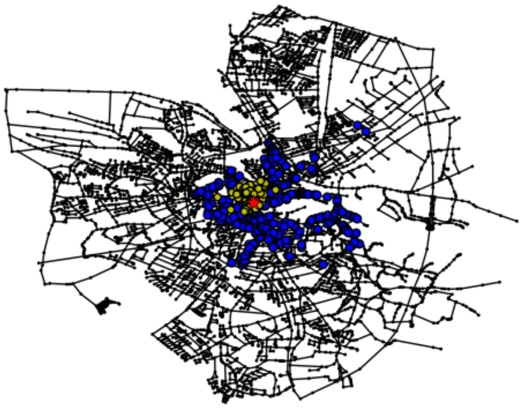

(b) $t = 2$

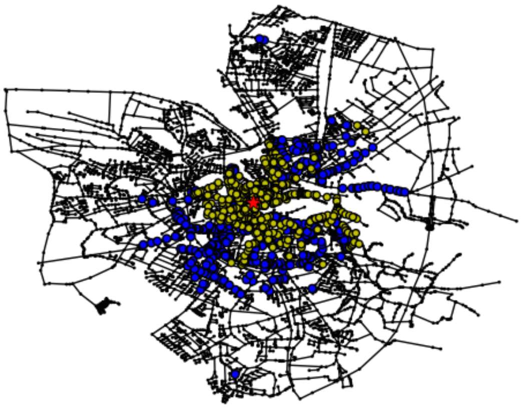

(c) $t = 3$

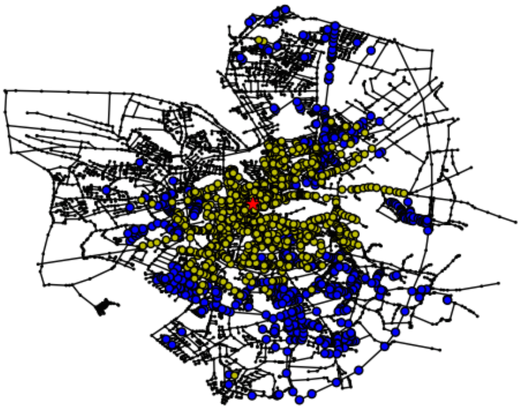

(d) $t = 4$

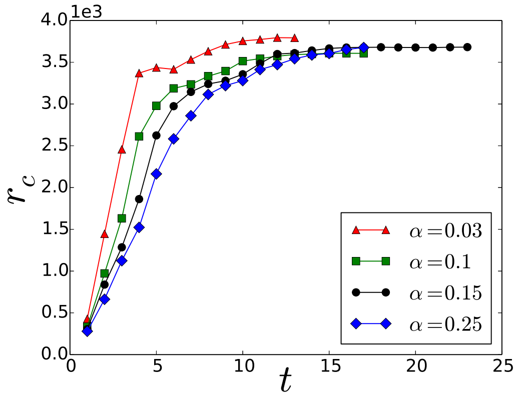

(e) radius of overloads

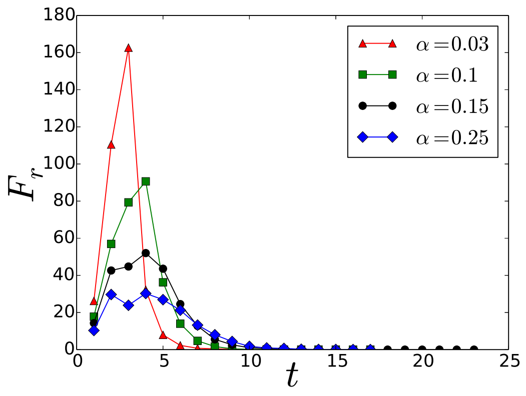

(f) number of failures

Supplementary Figure 5: **Overloads propagation in the road network of Oldenburg.** (a) to (d) are snapshots of overloads propagation at the first four steps on Oldenburg road network. Here we set $\alpha = 0.1$, $\sigma = 0.1$. The cascading overload failures are triggered by the initially removed 6 nodes in the central region. Green nodes stand for the survived nodes, red nodes stand for the initially attacked ones, blue nodes stand for the overloaded ones in the corresponding step, while yellow nodes stand for the overloaded ones in the previous steps. (e) and (f) are plots of $r_c(t)$ and $F_r(t)$.

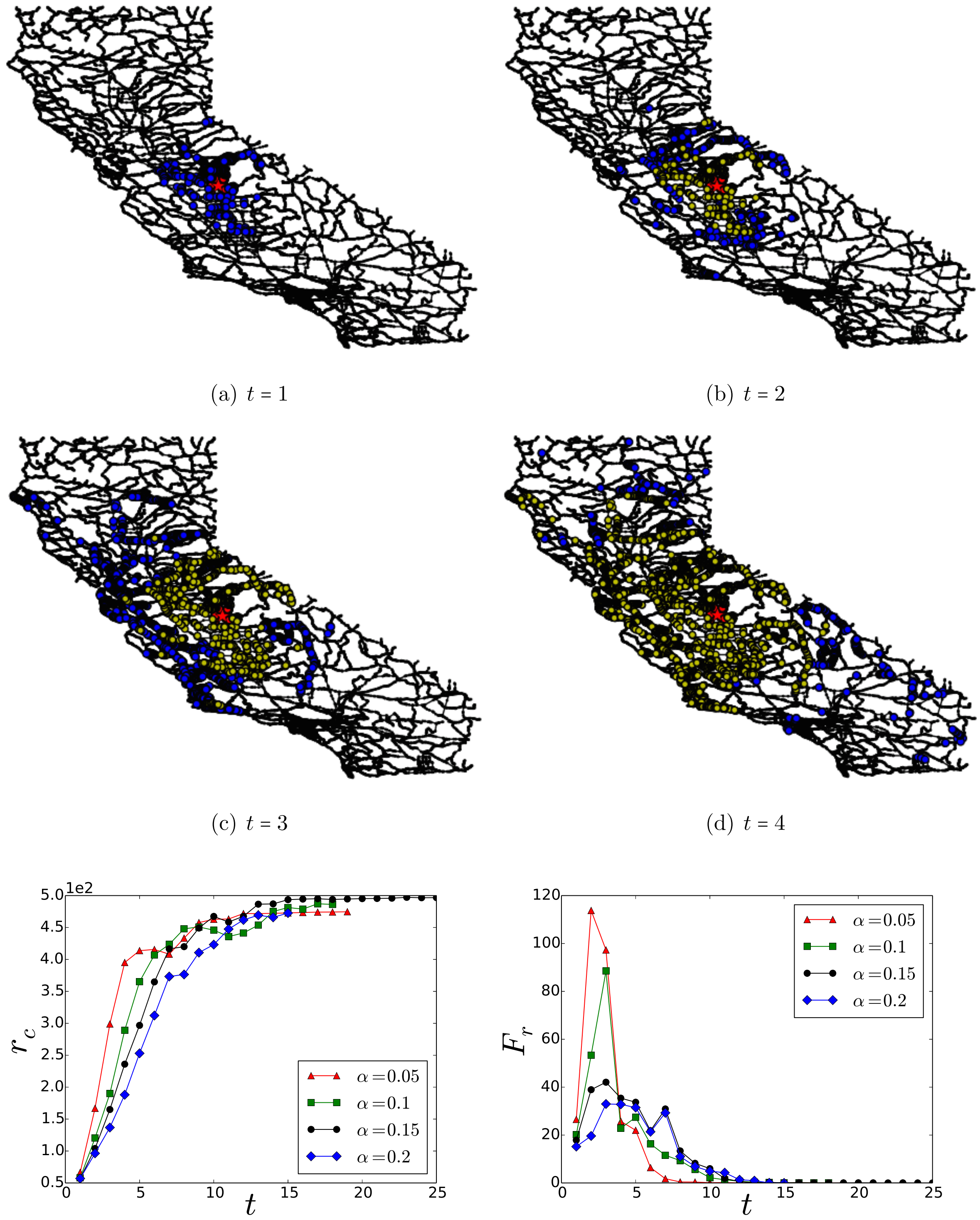

(a) $t = 1$

(b) $t = 2$

(c) $t = 3$

(d) $t = 4$

(e) radius of overloads

(f) number of failures

Supplementary Figure 6: **Overloads propagation in the road network of California.** (a) to (d) are the first four-steps of failure propagation in the road network of California. Here the initial failure is induced by removing 60 nodes in the geographical center of the network, which is denoted by the red star. Weights with Gaussian disorder ($\sigma = 0.1$) are imposed on the links and the tolerance is set to $\alpha = 0.025$. Behaviors of $r_c(t)$ and $F_r(t)$ are shown in (e) and (f).

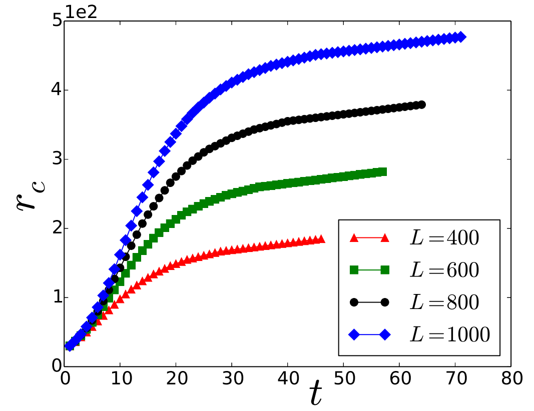

(a) $r_c(t)$

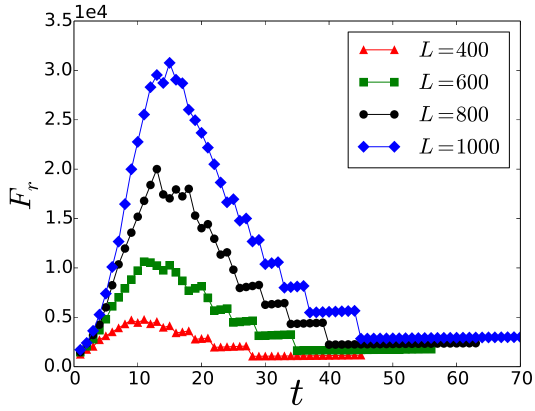

(b) $F_r(t)$

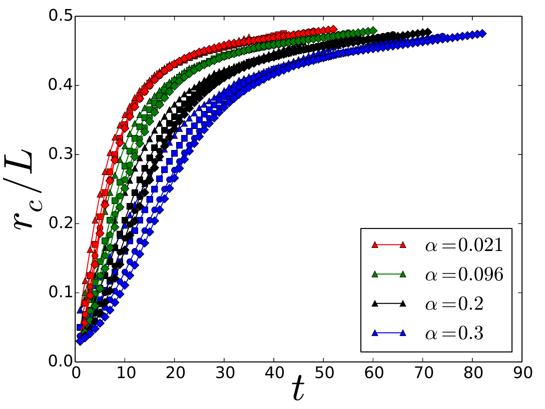

(c) $r_c(t)/L$

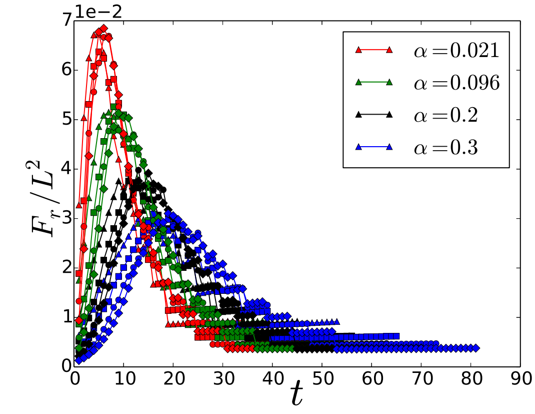

(d) $F_r(t)/L^2$

Supplementary Figure 7: **Results of the analytical model for large initial failure.** Here, the absolute radius of the initial failure is set to be $H = 30$ and the tolerance is set to be $\alpha = 0.2$. $L = 2R$ is the system linear size. In (c) and (d), different symbols stand for different resolutions ($1/R$), including 1/200 (△), 1/300 (□), 1/400 (○) and 1/500 (◇). Note that in theory F_r/L^2 still represents the fraction of failed nodes in each instant and its value is calculated through $\frac{F_r}{\pi R^2}$ instead of $\frac{F_r}{4R^2}$.

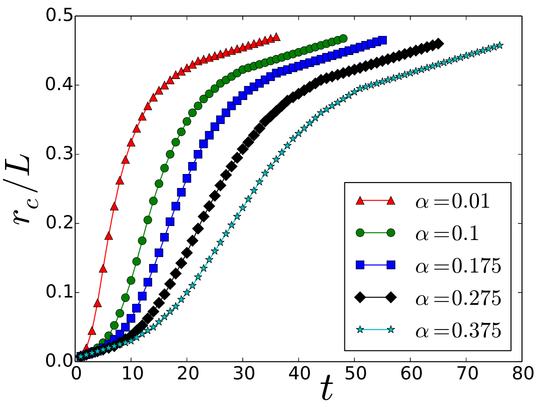

(a) $r_c(t)/L$ for different α

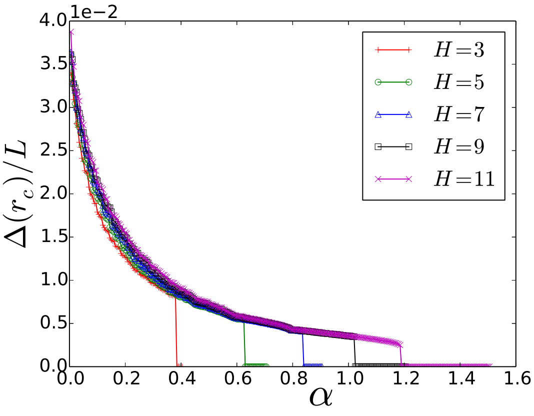

(b) $\Delta(r_c)$ of different initial damage sizes

Supplementary Figure 8: **The average velocity of the linear regime ($\Delta(r_c)$) approaches zero when $\alpha > \alpha_c$ in theory.** Here we set $L = 400$. In (a), H is set to 3.

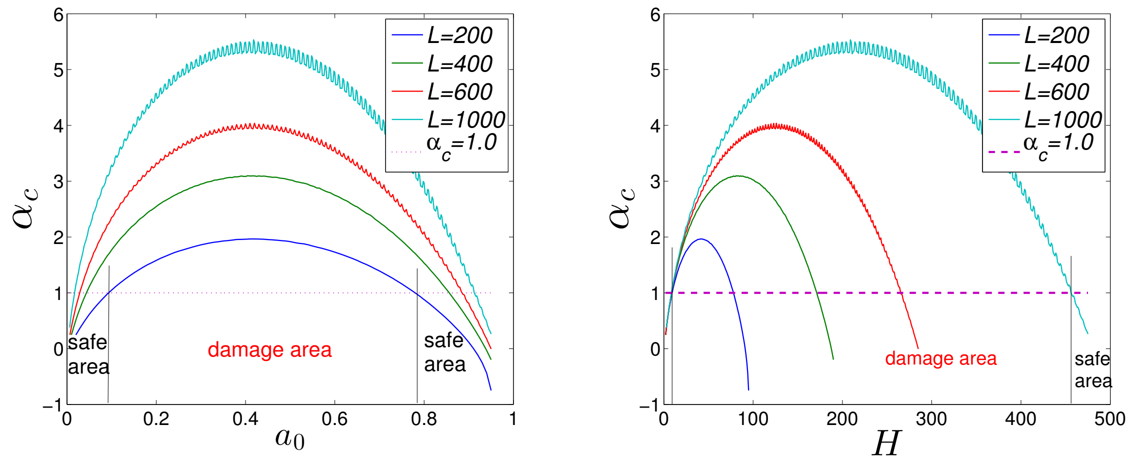

(a) (b)

Supplementary Figure 9: **The critical tolerance varies with radius of the initial failure in theory.** (a) α_c varies with the relative radius a_0 of the initial failure. (b) α_c varies with the absolute radius H of the initial failure. In fact, we can also get the critical attack size from this figure. Taking $L = 200$ with tolerance 1.0 as an example, we can split the range of initial failure radius into three regions. In the first region, the initial failure size is too small to trigger a cascade, in the middle one, there would be a cascade because the tolerance is not enough to protect the system, while in the last region, the initial damage is too large and the left part of the system would not be overloaded. Besides, as can be seen in (b), the critical tolerance, α_c, is almost the same for different resolutions as the initial failure size is extremely small.

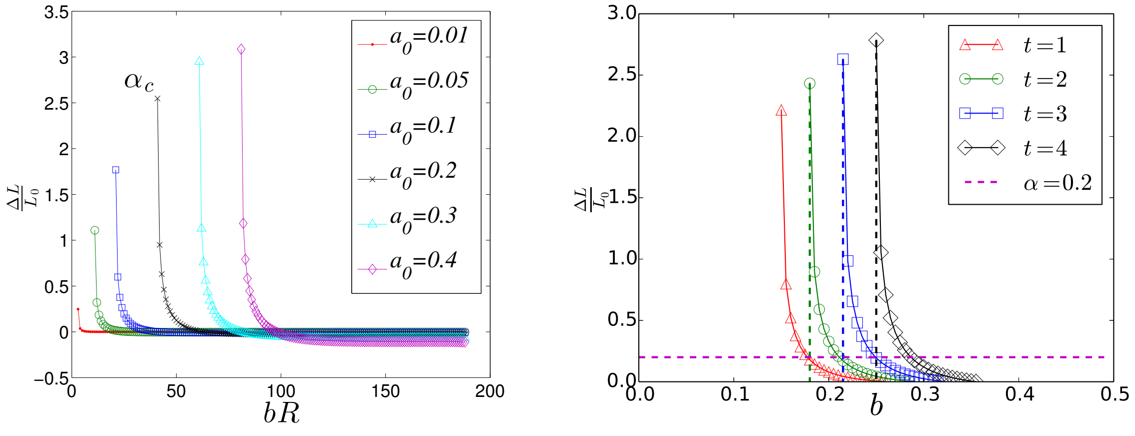

(a) (b)

Supplementary Figure 10: **Overloads as a function of distance in the analytical model, using Eq. 32 and Eq. 33.** (a) shows how $\frac{\Delta L}{L_0}$ varies with bR using the theory with resolution 1/200. Here a_0 is the relative radius of the initial failure, $\frac{\Delta L}{L_0}$ is the ratio between the overload and the initial load for the nodes located at distance bR from the system center O. Note that the overload at bR is calculated only at the initial stage due to the initial failure ($t = 1$). As seen, larger a_0 causes more overloads and possesses larger $\frac{\Delta L}{L_0}$. The maximum values of $\frac{\Delta L}{L_0}$ represent the values of α_c, and if $\alpha > \alpha_c$, no cascading overload failures will occur. (b) shows how $\frac{\Delta L}{L_0}$ varies with b at the first four steps in theory with resolution 1/200 when $\alpha = 0.2$ and $a_0 = 0.15$.

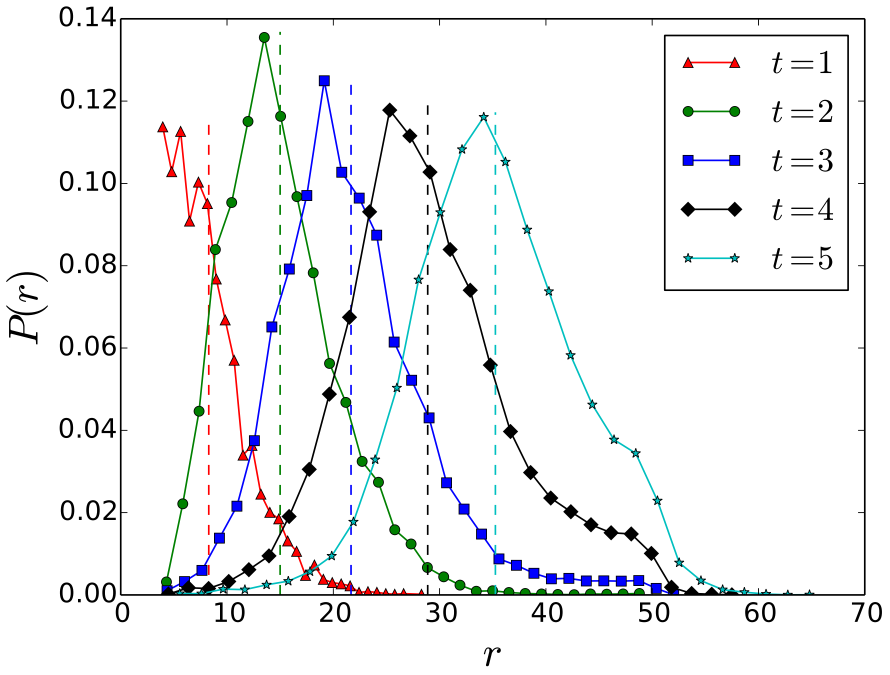

Supplementary Figure 11: **The spatial distribution of overloads in the simulation at different time steps during the overload cascading process.** For a given step, the distribution is found to have a sharp peak close to a characteristic radius $r_c(t)$ (marked with dashed line in the same color). The outliers are found negligible far away from this characteristic distance. The tolerance in this figure is α = 0.25.

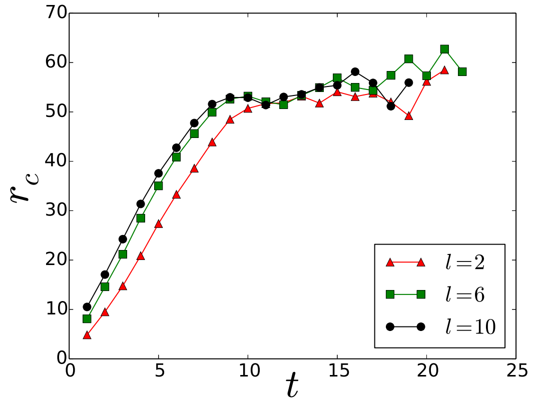

(a) Simulation with $\alpha = 0.25$

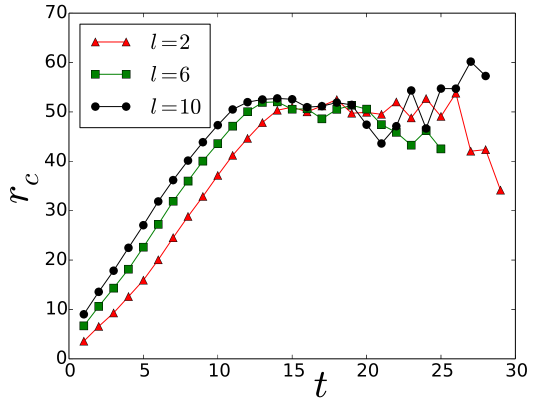

(b) Simulation with $\alpha = 0.5$

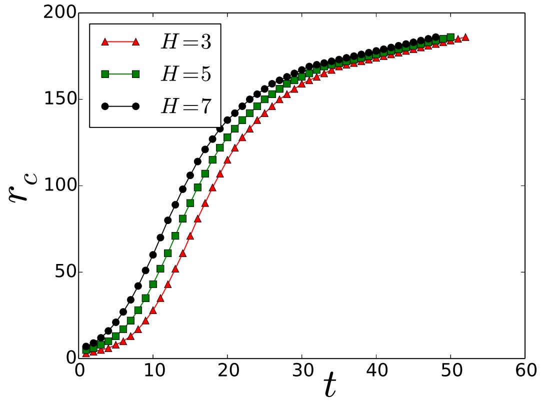

(c) Theory with $\alpha = 0.15$

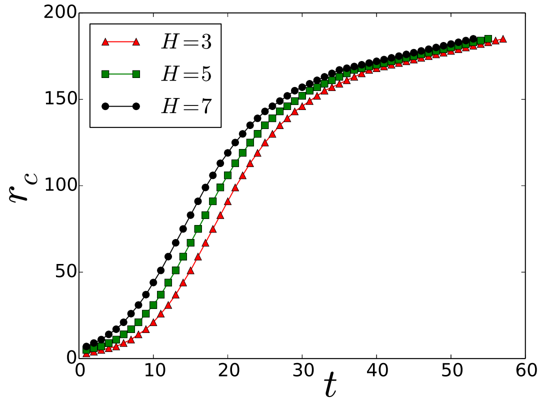

(d) Theory with $\alpha = 0.2$

Supplementary Figure 12: **The effects of initial damage on the overloads propagation velocity.** The plots show how r_c increases with t for different initial failures. In (a) and (b), we set $L = 100$ and $\sigma = 0.1$. Note that l is the linear size of the initial damage. The tolerance α is set to (a) 0.25 and (b) 0.5 in simulations, which are lower than their critical tolerances. In (c) and (d), we set $L = 400$. The tolerance is also smaller than the critical value, i.e., $\alpha = 0.15$ in (c) and $\alpha = 0.2$ in (d).

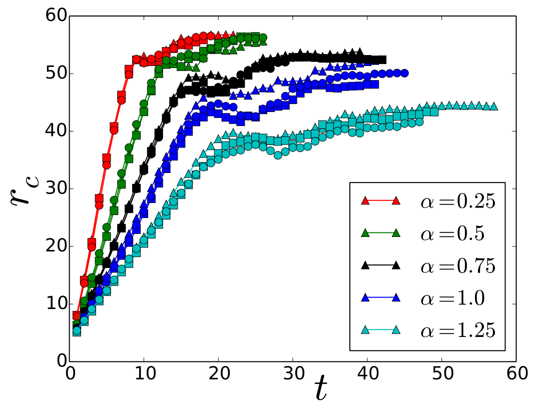

(a) Weighted lattice with $L = 100$

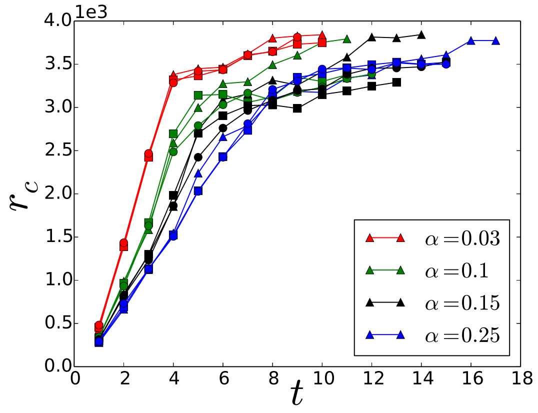

(b) Road network of Oldenburg

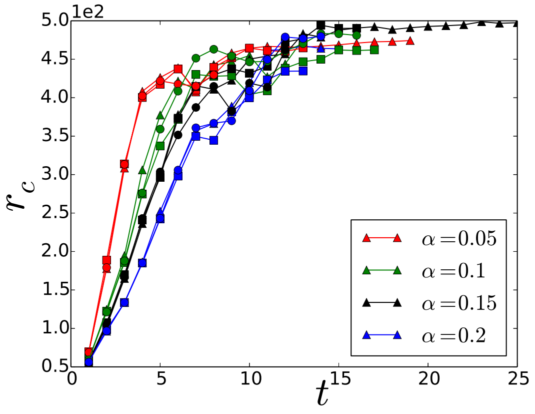

(c) Road network of California

Supplementary Figure 13: **The effects of disorder on the overloads propagation velocity.** It is shown how r_c varies with t for different disorders on (a) weighted lattice, (b) the road network of Oldenburg and (c) the road network of California. The $\triangle$ lines represent $\sigma = 0.1$ (mean is 1.0), the $\square$ lines represent $\sigma = 20.0$ (mean is 100.0) and the $\circ$ lines represent $\sigma = 2000.0$ (mean is 10000.0).

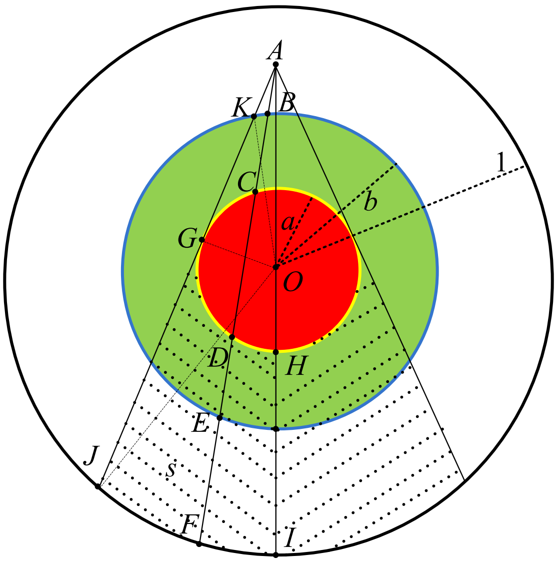

Supplementary Figure 14: **Modeling the extra load when $r > b$.** The network is embedded in a 2d circular plate centered at O with radius 1. The initial damage is located in the center of the network with radius $a \leq 1$ (red). The green ring centered at O and between a and b ($b > a$) is defined as the adjacent ring. The case of $r < b$ can be treated in a similar way.

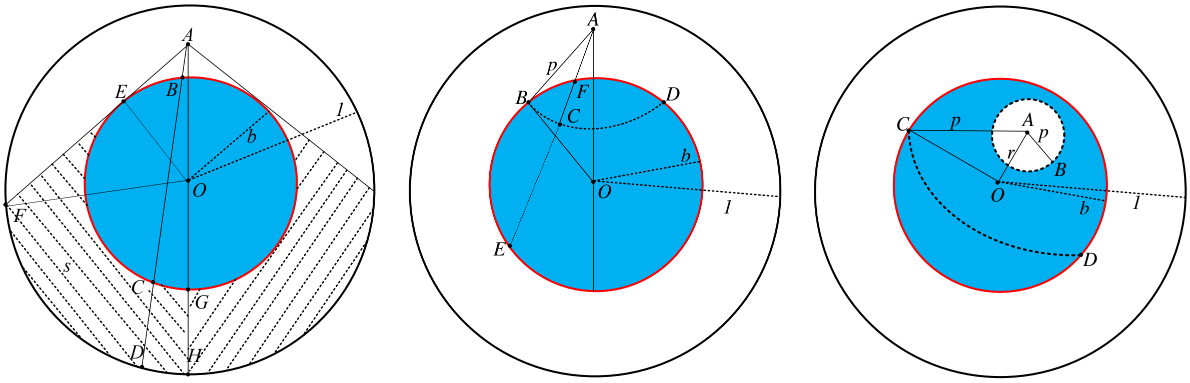

(a) *out – out* (b) *out – in* (c) *in – in*

Supplementary Figure 15: **Modeling the initial load.** Different possible cases of the analytical model are demonstrated in (a), (b) and (c) respectively, where $\mathfrak{B}$ is shown as a blue plate with radius b inside the system. O is the system center and the system radius is assumed to be 1. The radius of $\mathfrak{B}$ is $b \leq 1$.

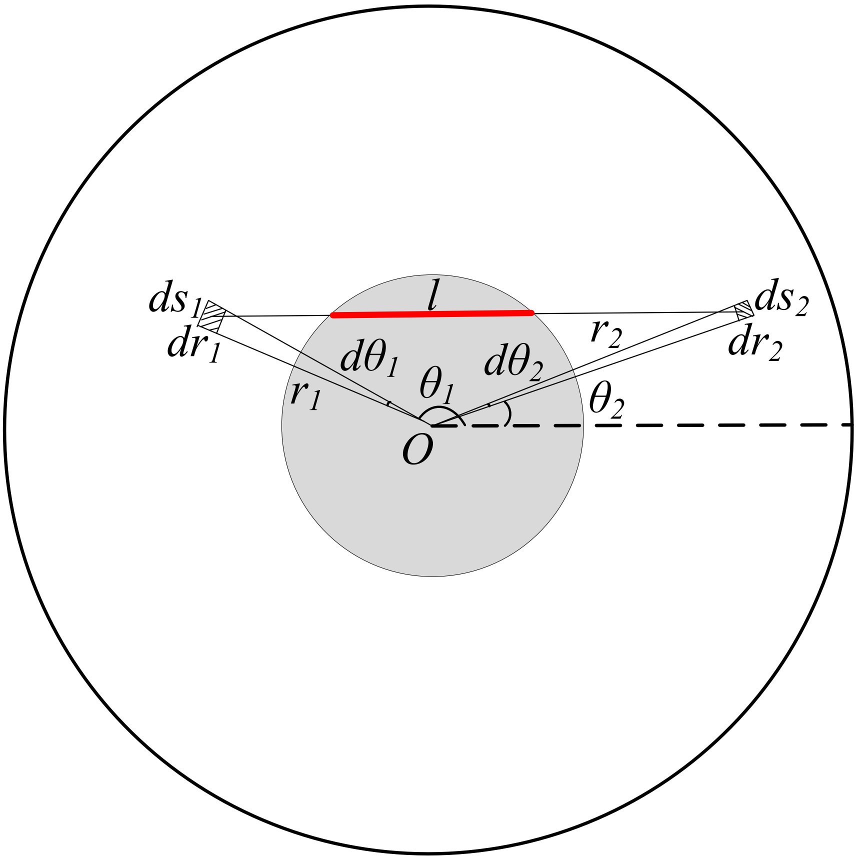

Supplementary Figure 16: **Illustration of the basic concept for the simplified theory.**

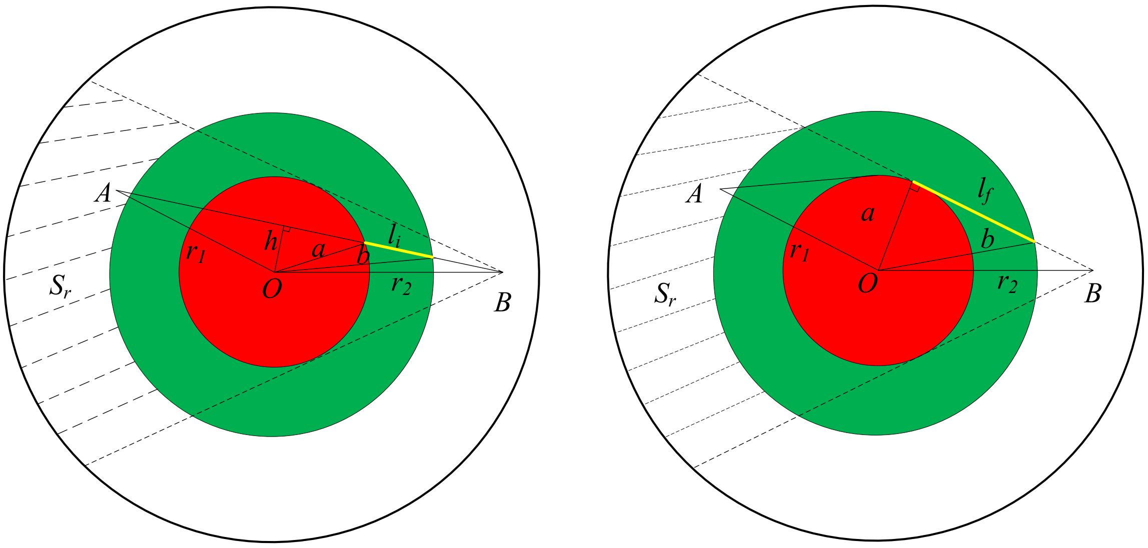

(a) Before the attack ($r_2 > b$) (b) After the attack ($r_2 > b$)

Supplementary Figure 17: **Illustration of the way to calculate the overload due to the regional attack.** Similarly to the previous framework, the red plate with radius a stands for the attacked area and the green ring with radius b is the area neighboring to the attack, whose nodes might be overloaded in the following cascades. Starting from a random node A with radius r_1, a path can arrives at node B (with radius r_2) on the other side by passing through the ring or the attacked area. In (a), the path between A and B can directly pass though the red plate and its length inside the green ring is l_i (yellow). In (b), because of the attack, the path from A has to walk along the tangent line of the attacked area before arriving at B, hence its length inside the green ring will be changed to l_f (yellow). Actually, increment of the path length due to the attack will impose overload to the green ring.

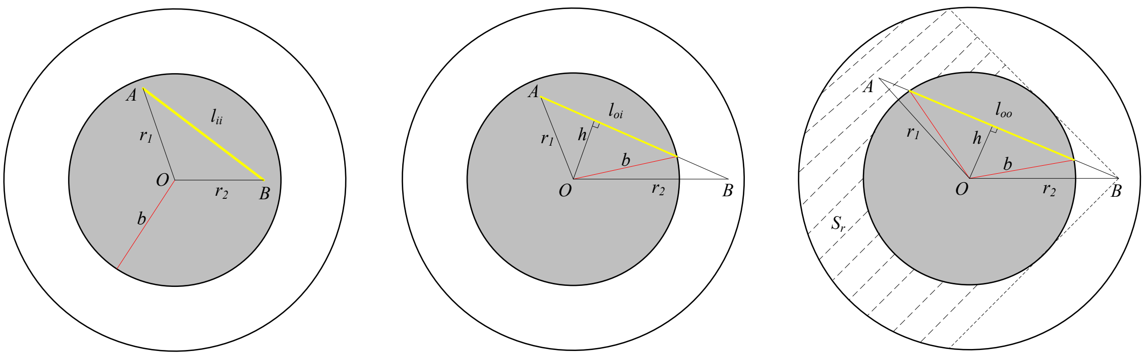

(a) The case of $in - in$ (b) The case of $out - in$ (c) The case of $out - out$

Supplementary Figure 18: **Illustration of how to calculate the initial load of the system.**

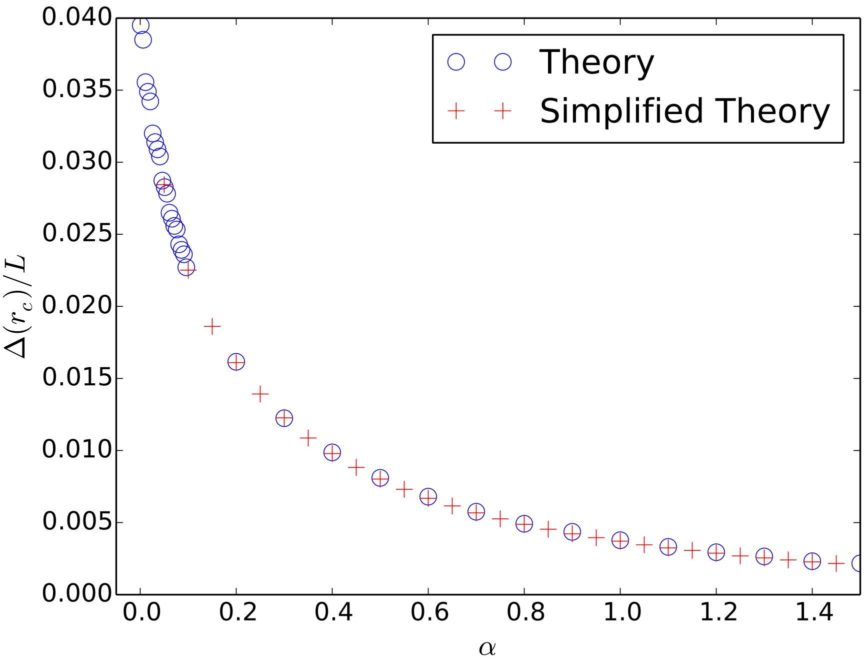

Supplementary Figure 19: **Comparison between the original theory and the simplified one.** Here we set $L = 2R = 1000$, $H = 30$, $\Delta(r_c)$ is the averaged velocity of the linear regime in failure propagation.

Supplementary Notes

Supplementary Note 1: Propagation of overloads on other model networks

Here we also report the dynamics of failure propagation on other model networks, including weighted circular lattice, weighted planar graph and weighted circular plate. The local propagation behavior in these model networks is also found to have similar velocity as in the weighted lattice and other real networks (Fig. 5(a) in the paper).

Weighted circular lattice

For a lattice without periodic boundaries, we get a circular lattice by excluding the nodes with radius (Euclidean distance to the center of the system) larger than $L/2$, where L is linear size of the lattice. The network links are allocated with Gaussian weights. Similarly, we attack the obtained circular lattice by removing nodes in the center with radius smaller than $l/2$, where l is defined as the linear scale of the attack. The results of failure propagation are demonstrated in Supplementary Figure 1. In Supplementary Figures 1(a)-1(d), red dots are the initially attacked nodes, blue dots are the nodes overloaded at the corresponding step, black dots stand for the nodes that failed in the previous step and green dots represent the functional survivals.

Weighted planar graph

For the planar graph, we uniformly get coordinates x_i and y_i from $(0, 1)$ for each node i and then the Delaunay triangularization [1] is used to connect different nodes. The planar graph is also disordered with Gaussian weights on links, and nodes within the central $l \times l$ region are removed to trigger the cascading failures. The dynamics of failure propagation are demonstrated in Supplementary Figure 2. Note that in Supplementary Figures 2(a)-2(d), red nodes are the initially attacked ones, blue nodes are the ones failed at the corresponding step, yellow nodes represent the previously overloaded ones and green nodes are the functional survivals.

Weighted circular plate

A circular plate is also designed to test the overload propagation on network with a different shape. The circular plate network is composed of a central node and $L/2$ layers, where L is the diameter of the system. On each layer $l_a \geq 1$, we place $6l_a$ nodes and connect them by a loop with radius l_a. For links between neighboring layers, as can be seen in Supplementary Figure 3, the central node 0 (red) will be connected to all the nodes in layer 1, and for other layers ($l_a \geq 1$), each node in layer l_a will be linked to the nodes regularly in layer $l_a + 1$ except the backbones (lines starting from the central node, like $0 - 1 - 7 - 19$).

Similarly, we put Gaussian weight with $\sigma = 0.1$ on each link in the circular plate. The central node, and the first layer (total 7 nodes) are removed to trigger the following cascades. The dynamics of the failure propagation can be found in Supplementary Figure 4, and the behaviors of $r_c(t)$ and $F_r(t)$ are also consistent with results found on other models.

Supplementary Note 2: Cascading failures on realistic networks

In order to test our results on real networks, we performed simulations on the realistic road network [2] in the city of Oldenburg and California. The road network of Oldenburg has 6105 nodes and 7029 edges, and the road network of California has 21048 nodes and 21693 edges. The velocity of overload propagation is found similar as in the simulation on model networks (Fig. 5(a) in the paper).

As shown in Supplementary Figure 5, after the initial local attack on the network center of road network in Oldenburg, the behavior of r_c and F_r in Supplementary Figures 5(e) and 5(f) is similar to that seen in theory and simulations.

Another example of failure propagation in the realistic road network of California is demonstrated in Supplementary Figure 6. The cascading patterns observed in Supplementary Figures 6(e) and 6(f) on this network are similar to our results on weighted lattice and theory.

Supplementary Note 3: Results from the analytical model

In Fig. 4 of the paper, we presented the analytical results of the model, $r_c(t)$, which show a large range of linear slope but a slow increase at first few steps when the initial failure is very small. In Supplementary Figure 7, we analyze the case when the absolute radius of the initial failure is larger, e.g., $H = 30$, and find that the measures show a fast and almost constant rate of growth with time already in the first few steps, in close agreement with simulation. We also show in Supplementary Figures 7(c) and 7(d) that the scaling with system size is valid.

Since the theoretical model is not constrained by the complexity in simulations of finding optimal paths, it greatly simplifies the way of calculating the critical damage size and the critical tolerance, which is denoted as α_c. Specifically, if the value of tolerance is higher than the threshold, there would be no cascading overload failures. Hence for different initial failures with relative radius a_0, we observe in Supplementary Figure 10 how $\frac{\Delta L}{L_0}$ decreases as b increases using Eq. 32 and Eq. 33. As can be seen in Supplementary Figure 10(a), we find that $\frac{\Delta L}{L_0}$ decreases fast with increasing bR. It indicates that as b increases, the nodes on the circle with radius b suffer declining overloads. Moreover, for each a_0, the maximum of $\frac{\Delta L}{L_0}$ is the critical tolerance, α_c. Accordingly, in each step of the failure propagation, as shown in Supplementary Figure 10(b), $\frac{\Delta L}{L_0}$ decreases as b increases. Since the damage size for each step continues to expand when the failure spreads, the maximum $\frac{\Delta L}{L_0}$ also grows with t.

We also investigate the critical tolerance as a function of the relative size (a_0) and absolute size (H) of initial damage respectively based on theory. As shown in Supplementary Figure 9, α_c increases with the size of the initial damage and because of the finite size effect, it starts to decrease when the relative linear size of the damage is approximately half of the system size. With regard to the spreading velocity, it changes abruptly from a finite value below α_c to zero above α_c for finite system sizes in the theory. As can be seen in Supplementary Figure 8(a), $\alpha_c \approx 0.38$ for $L = 400$ and $H = 3$, but the failure can still propagate to the border of the system even for $\alpha = 0.375$. Besides, as shown in Supplementary Figure 8(b), the average velocity of the linear regime ($\Delta\langle r_c\rangle$) jumps to zero abruptly as α approaches the critical value for different sizes of initial damage, and the larger initial damage size requires higher α_c.

Supplementary Note 4: The spatial distribution of overload failures

We have studied in the simulation the spatial distribution of overload failures at each time step, and found in Supplementary Figure 11, that the radius (distance to the center of initial damage) of these overloads at each time step is distributed with a sharp peak at a characteristic distance, with rare outlier events away from this characteristic distance. This occurs due to the redistribution of optimal paths close to the previous damage region, in spite of global interactions. In this redistribution, the new optimal paths, which previously passed the damage area, need to surround the damage through its neighboring region. Meanwhile, our findings are also supported by the statistics from model [3] and realistic outages [4], where the probability of failures is also found decreasing sharply from a characteristic distance.

The existence of this characteristic distance supports our definition of an average radius $r_c(t)$, which can help to predict the collective propagation of overloads. Note that the behavior of outliers, in spite of their rareness, is worth to study in the future, in order to fully capture the cascading overloads.

Supplementary Note 5: The effects of the initial damage

As seen in Supplementary Figure 10(a) the size of the initial failure does influence α_c, since more damage can yield more overloads. It seems, however, that the velocity of the cascading overload failures is not affected by the initial damage as long as the tolerance is lower than the critical value. As shown in Supplementary Figure 12, in both simulation and theory, $r_c(t)$ grows linearly with t with almost same slope for different initial failure sizes. Moreover, this finding is consistent with Supplementary Figure 8(b), in which the velocity curves of different initial damage sizes overlap together when α is smaller than the corresponding α_c.

Supplementary Note 6: The effects of the disorder

In the simulations on weighted lattice and realistic systems, we allocate weight to each link of the network, which is chosen from a Gaussian distribution with variance σ. Hence except the initial damage, we also investigate the effects of the disorder by tuning σ. However, simulations with different σ suggest that the disorder in certain range does not significantly affect the propagation velocity of the cascading overload failures. In Supplementary Figure 13, overlapping r_c curves for different disorders are shown on both weighted lattice and realistic networks.

Supplementary Methods

The analytical model for cascading overloads

The propagation of overloads is caused by redistribution of load in the network, because the initial failure can induce the increase of shortest paths that are now passing through the remaining functional nodes. Besides, from the overload propagation simulations (Fig. 1 of the paper) on weighted lattice, we can learn that the cascading overloads show an approximately radial diffusion from the center of the initial failure. Based on these observations, we develop an analytical framework to model the propagation of overload failure in spatially embedded networks.

Modeling and evaluating the extra load

The essence of the cascading overloads is that the remaining functional nodes would suffer extra load, which might crash in the next step due to their limited tolerance. Here we denote the extra load as ΔL_r and first discuss how to model it.

Given the radial diffusion of the overload failures, we simply assume that the network is embedded in a $2d$ circular plate centered at O with radius 1, as shown in Supplementary Figure 14 (we replot Fig. 3 of the paper here in order to better demonstrate the analytical model). Similarly, we let the initial failure happen in the center of the network, within a circle centered at O with radius $a \leq 1$ (red circle in Supplementary Figure 14). The nodes within the initial failure area are removed, and then the nodes nearby, in the green ring centered at O between a and b ($b > a$), will suffer extra load because of the initial failure. We call this ring the adjacent ring, and if the extra load induced exceeds the tolerance of these nodes, then the nodes in this ring will fail in the next step, indicating the overload failure cascades forward. Therefore, the problem of modeling extra load can be converted to how to estimate the overload on the adjacent ring.

As can be seen in Supplementary Figure 14, we define a random node A in the network, whose distance from O is $r \leq 1$ and AF is a path starting from A to a random node F on the border of the system. As $r > b$, the intersection points between AF and the circle with radius b are B and E, respectively. It also meets the border of the failure area at C

and D. While AJ is a tangent line to the failure area, which starts from A, ends at J and the tangent point is G. We also define another path AI, which starts from A to a border node I by passing through the system center O. Since the nodes within the failure area do not function, shortest paths starting from A to destinations located in the shadow s (the dotted area in Supplementary Figure 14) would be affected and become longer. Specifically, the first part of these shortest paths would pass through the adjacent ring along AG with length $\sqrt{b^2 - a^2}$ (Note that when $r \leq b$, its length changes to $\sqrt{r^2 - a^2}$). Without initial failure, these paths could pass through the failure area directly and the length of their first parts within the adjacent ring should be BC, for example. Therefore, the extra load that the adjacent ring suffers is reflected by the increased length of shortest paths within the adjacent ring after the failure happens.

In order to simplify the analysis, we let $AB = y$, $AC = x$, $AF = c(1)$, the angle between AG and AO will be β and the angle between AC and AO is θ. Obviously, we have $\beta = \arcsin \frac{a}{r}$.

As $r > b$, A is located outside the adjacent ring and we have,

$$\begin{aligned} x^2 + r^2 - 2xr\cos\theta &= a^2, \\ y^2 + r^2 - 2yr\cos\theta &= b^2. \end{aligned} \tag{1}$$

For $x \leq r\cos\theta$ and $y < r\cos\theta$, we get the solutions as

$$\begin{aligned} x &= r\cos\theta - \sqrt{a^2 - r^2\sin^2\theta}, \\ y &= r\cos\theta - \sqrt{b^2 - r^2\sin^2\theta}. \end{aligned} \tag{2}$$

Similarly, we can obtain

$$c(1) = r\cos\theta + \sqrt{1 - r^2\sin^2\theta}. \tag{3}$$

Then, we get BC, which is also the length of the first part (within the adjacent ring) of the shortest paths from A to the failure area and the shadow area. Here we define it as

$$l_1(\theta) = x - y = \sqrt{b^2 - r^2\sin^2\theta} - \sqrt{a^2 - r^2\sin^2\theta}. \tag{4}$$

When $r < b$, i.e., A is located inside the adjacent ring and similar to the above analysis, we can obtain the length of the first part of shortest paths as

$$l_1(\theta) = r\cos\theta - \sqrt{a^2 - r^2\sin^2\theta} \tag{5}$$

Note that when $r = b$, Eqs. 4 and 5 are equivalent, hence we can rewrite them together as

$$l_1(\theta) = \begin{cases} r\cos\theta - \sqrt{a^2 - r^2\sin^2\theta}, & r \le b \\ \sqrt{b^2 - r^2\sin^2\theta} - \sqrt{a^2 - r^2\sin^2\theta}, & r > b \end{cases} . \tag{6}$$

Note that the number of shortest paths also varies with θ, which could be easily evaluated as

$$l_2(\theta) = c(1) - x. \tag{7}$$

Thus, the average length of the first part within the adjacent ring in the absence of failures can be written as

$$v(a,b,r) = \frac{\int_0^\beta l_1(\theta) l_2(\theta) d\theta}{\int_0^\beta l_2(\theta) d\theta}. \tag{8}$$

The number of A's destinations within the shadow can be represented by the area of the shadow regime, which is

$$s(a,r) = (\pi - \phi - \varphi)(1 - a^2) + a\sqrt{1 - a^2} - \varphi a^2, \tag{9}$$

where $\phi = \arccos\frac{a}{r}$ and $\varphi = \arccos a$.

Therefore, after the initial failure, the extra load imposed on the adjacent ring by the first part of the shortest paths starting from A is $\sqrt{r^2 - a^2}s(a,r) - v(a,b,r)(s(a,r) + \pi a^2)$ for $a \le r \le b$ and $\sqrt{b^2 - a^2}s(a,r) - v(a,b,r)(s(a,r) + \pi a^2)$ for $b \le r \le 1$. The second part of the paths could be included by moving A around the circle centered at O with radius r. Thus, the integration from $r = a$ to $r = 1$ yields all the overloads induced on the adjacent ring after the initial failure,

$$\Delta L_r(a,b) = \int_a^b [\sqrt{r^2 - a^2}s(a,r) - v(a,b,r)(s(a,r) + \pi a^2)] 2\pi r dr + \int_b^1 [\sqrt{b^2 - a^2}s(a,r) - v(a,b,r)(s(a,r) + \pi a^2)] 2\pi r dr. \tag{10}$$

Note that ΔL_r is a function of a and b.

Modeling and evaluating the initial load

In the above section we presented a method to estimate the extra load induced on the adjacent ring due to the initial failure within a circle of radius a. Following a similar approach, an estimation of the initial load is presented in this section. As seen in Supplementary

Figure 15, we assume a circular plate $\mathfrak{B}$ (the blue area of radius b in Supplementary Figure 15) inside the network and model its load by calculating the amount of flow through it. We define a random node A in the network and its distance to the center O is $r \le 1$. Then, the shortest paths starting from A to other nodes of the system could be divided into three cases:

- *out − out* (*oo*): $b < r \le 1$, A is located outside $\mathfrak{B}$ and its destinations are also located outside $\mathfrak{B}$. The shortest paths passing through $\mathfrak{B}$ are shown in Supplementary Figure 15(a).
- *out − in* (*oi*): $b < r \le 1$, A is located outside $\mathfrak{B}$, while its destinations are located inside $\mathfrak{B}$, and their shortest paths start from A and end at a random node in $\mathfrak{B}$ (e.g., C), as shown in Supplementary Figure 15(b).
- *in − in* (*ii*): $r \le b$, A is located inside $\mathfrak{B}$ and its destinations are also located inside $\mathfrak{B}$, which means the shortest paths are located inside $\mathfrak{B}$, as shown in Supplementary Figure 15(c).

The case *out − out*. In order to simplify the analysis, as can be seen in Supplementary Figure 15(a), we let $AB = x$, $AC = c(b)$ and $AD = c(1)$. AE is the tangent path starting from A through the circle $\mathfrak{B}$, the angle between AE and AO is $\beta = \arcsin \frac{b}{r}$ and the angle between AB and AO is θ. As shown in Supplementary Figure 15(a), the length of the parts within $\mathfrak{B}$ of the shortest paths starting from A to the shadow s of $\mathfrak{B}$ is BC. We have

$$\begin{aligned} x^2 + r^2 - 2xr\cos\theta &= b^2, \\ c(b)^2 + r^2 - 2c(b)r\cos\theta &= b^2. \end{aligned} \tag{11}$$

And since $x \le r\cos\theta$ and $c(b) \ge r\cos\theta$, we get the solutions as

$$\begin{aligned} x &= r\cos\theta - \sqrt{b^2 - r^2\sin^2\theta}, \\ c(b) &= r\cos\theta + \sqrt{b^2 - r^2\sin^2\theta}. \end{aligned} \tag{12}$$

Similarly we have

$$c(1) = r\cos\theta + \sqrt{1 - r^2\sin^2\theta}. \tag{13}$$

Therefore, the length within $\mathfrak{B}$ is

$$l_1^{oo}(\theta) = c(b) - x = 2\sqrt{b^2 - r^2\sin^2\theta}, \tag{14}$$

while the number of the shortest paths can be written as

$$l_2^{oo}(\theta) = c(1) - c(b) = \sqrt{1 - r^2 \sin^2 \theta} - \sqrt{b^2 - r^2 \sin^2 \theta}. \tag{15}$$

Hence the average length of the shortest paths within $\mathfrak{B}$ is

$$v^{oo}(b, r) = \frac{\int_0^\beta l_1^{oo}(\theta) l_2^{oo}(\theta) d\theta}{\int_0^\beta l_2^{oo}(\theta) d\theta}. \tag{16}$$

The number of A's destinations can be evaluated by the area of the shadow s (the dashed area in Supplementary Figure 15(a)),

$$s(b, r) = (\pi - \phi - \varphi)(1 - b^2) + b\sqrt{1 - b^2} - \varphi b^2, \tag{17}$$

where $\phi = \arccos \frac{b}{r}$ and $\varphi = \arccos b$. According to Eq. 10, we can get the flux produced by the shortest paths between the pairs of nodes outside $\mathfrak{B}$ as

$$L_{ini}^{oo} = \int_b^1 \pi r v^{oo}(b, r) s(b, r) dr. \tag{18}$$

The case *out – in*. As shown in Supplementary Figure 15(b), we let $AF = y$ and BD be an arc with center A and radius p, where $r - b < p < r + b$. Then AC is the shortest path from A to nodes inside $\mathfrak{B}$ (C is on BD) and the length of the part within $\mathfrak{B}$ is FC. As p increases, BD would eventually cover all the nodes inside $\mathfrak{B}$ and we can then get the flux from A to them. We denote the angle between AB and AO as β, which is a function of p, b and r,

$$\beta = \arccos \frac{p^2 + r^2 - b^2}{2pr}. \tag{19}$$

Let the angle between AF and AO be θ, then similarly to Eq. 12 we have

$$y = r \cos \theta - \sqrt{b^2 - r^2 \sin^2 \theta}. \tag{20}$$

Therefore, the average length of the parts within $\mathfrak{B}$ for the shortest paths from A to nodes on BD can be written as

$$l_1^{oi}(p) = \frac{\int_0^\beta (p - y) d\theta}{\beta} = p - \frac{\int_o^\beta y d\theta}{\beta}. \tag{21}$$

And the number of paths is

$$l_2^{oi}(p) = \frac{2\beta}{2\pi} 2\pi p = 2\beta p. \tag{22}$$

Then the average length of the shortest paths within $\mathfrak{B}$ is

$$v^{oi}(b, r) = \frac{\int_{r-b}^{r+b} l_1^{oi}(p) l_2^{oi}(p) dp}{\int_{r-b}^{r+b} l_2^{oi}(p) dp}. \tag{23}$$

Here the number of A's destinations is exactly the area of $\mathfrak{B}$, which is πb^2. The total flux imposed on $\mathfrak{B}$ by the shortest paths between A and the nodes inside $\mathfrak{B}$ is

$$L_{ini}^{oi} = \int_b^1 2\pi r \pi b^2 v^{oi}(b,r)dr. \tag{24}$$

The case *in* − *in*. As shown in Supplementary Figure 15(c), A and its destinations in this case are all located inside $\mathfrak{B}$. Let A be a center of a circle of radius $p > 0$ inside $\mathfrak{B}$. Then the shortest paths from A to the nodes on the circle or arc would impose flux to $\mathfrak{B}$ and their length is p. Different from the previous cases, here we need to discuss two situations. First, as $p \le b - r$, the circle centered at A is completely located within $\mathfrak{B}$ and the number of shortest paths is $2\pi p$. Second, as $b - r < p \le b + r$, the circle centered at A intersects with $\mathfrak{B}$ at points C and D, and the number of shortest paths is

$$\frac{2\beta}{2\pi} 2\pi p = 2\beta p, \tag{25}$$

where β is the angle between AC and AO,

$$\beta = \arccos \frac{p^2 + r^2 - b^2}{2pr}. \tag{26}$$

Hence the length of the shortest path is simply

$$l_1^{ii}(p) = p, \tag{27}$$

while the number of shortest paths can be written as

$$l_2^{ii}(p) = \begin{cases} 2\pi p, & 0 < p \le b - r \\ 2\beta p, & b - r < p \le b + r \end{cases}. \tag{28}$$

Therefore, the average length of the shortest paths within $\mathfrak{B}$ is

$$v^{ii}(b,r) = \frac{\int_0^{b+r} l_1^{ii}(p) l_2^{ii}(p) dp}{\int_0^{b+r} l_2^{ii}(p) dp}. \tag{29}$$

The number of destinations for A is still the area of $\mathfrak{B}$, then the total flux generated by the shortest paths starting from A to nodes within $\mathfrak{B}$ is

$$L_{ini}^{ii} = \int_0^b \pi r v^{ii}(b,r) \pi b^2 dr. \tag{30}$$

Finally, by adding all the three cases together, we can obtain the total load imposed on $\mathfrak{B}$

$$L_{ini}(b) = L_{ini}^{oo} + L_{ini}^{oi} + L_{ini}^{ii}, \tag{31}$$

which is a function of b.

Numerical solution of the analytical model

From the above theoretical model, a node on a circle centered at O with radius b would suffer the extra load

$$\Delta L = \frac{1}{2\pi b}\frac{\partial}{\partial b}\Delta L_r(a,b) \tag{32}$$

due to the failure within a radius $a < b$. While its initial load can be written as

$$L_0 = \frac{1}{2\pi b}\frac{\partial}{\partial b}L_{ini}(b). \tag{33}$$

Then, based on the limited capacity, assuming a tolerance α, the critical condition for a node to fail in the next step is

$$\begin{aligned}(1+\alpha)L_0 &= L_0 + \Delta L\\ \alpha &= \frac{\Delta L}{L_0}.\end{aligned} \tag{34}$$

When $\alpha \geq \frac{\Delta L}{L_0}$, nodes on the circle of radius b will not be overloaded and the failure will be stopped; however, if $\alpha < \frac{\Delta L}{L_0}$, all the nodes on this circle would crash and the failures will spread further.

In contrast to the heavy computations needed in the simulations, numerical solutions of the analytical model are not limited by the network size. Therefore, it is possible in the theory to disclose the patterns of cascading overloads on much larger systems compared to simulations. Furthermore, the analytical model also provides a convenient way to explore the interplay between the critical tolerance, the initial damage size and the linear size of the network. The details of the numerical solutions are as follows. First, we assume that the initial failure is within a radius $a(t=0) = a_0$ and the system tolerance is α. Besides, the resolution of the analytical model, denoted as $1/R$, is also a parameter, which determines the system size. Next, we start from analyzing Eq. 10 and Eq. 31 for $b = a(t) + 1/R$ to calculate the overloads in b at the next step, $t+1$. For a certain value of b, if $\alpha < \frac{\Delta L}{L_0}$, then the nodes within the adjacent ring would fail. Hence we let $b = b + 1/R$ and continue the search until we reach b^* where $\alpha \geq \frac{\Delta L}{L_0}$. It means that we have arrived the border of the failure in step t and the initial failure for step $t+1$ is updated by b^* to reiterate the load redistribution. If no b^* is found, it means that the given α is large enough to protect the network against the initial failure. Note that from the definition of the resolution, we consider the system linear size as $L = 2R$, which is the diameter of the circle. The

total number of nodes is πR^2 and the absolute radius of the initial failure is defined as $H = a_0 R$. The radius of the overloads at step t is actually $r_c(t) = a(t)R$ and similarly we have the failures at step t to be $F_r(t) = \pi(a^2(t) - a^2(t-1))R^2$.

The simplified theory

Methodology

A simplified theory is presented in this section and its basic concept is shown in Supplementary Figure 16. As can be seen, the load of the gray plate inside the system can be calculated by the product of length and number of paths passing through it. Assuming that nodes inside a extremely small area ds_1 (with polar coordinates r_1 and θ_1) share the same optimal path to the nodes locating in another small region ds_2 (with polar coordinates r_2 and θ_2) and the length inside the gray plate is l (red), the load imposed by those paths can be written as lds_1ds_2, where $ds_1 = r_1 d\theta_1 dr_1$ and $ds_2 = r_2 d\theta_2 dr_2$. We denote the possible origin area as S_1 and the possible destination area as S_2, and then the integration over both of the area will represent the total load L_t imposed on the gray plate, which can be written as

$$L_t = \iint_{S_2 S_1} l ds_1 ds_2. \tag{35}$$

Next, based on this methodology, we will calculate the overload of the survival nodes due to the attack, and the initial load of the network. Note that without loss of generality, we set $\theta_2 = 0$ in the following illustrations to ease the elucidation and $\int d\theta_2 = 2\pi$.

Modeling the overload

Before attack, when $r_2 > b$, as can be seen in Supplementary Figure 17(a), the path length inside the ring can be written as $l_i = \sqrt{b^2 - h^2} - \sqrt{a^2 - h^2}$, h is the distance from the center to the path and it can be written as $\frac{r_1 r_2 \sin\theta_1}{\sqrt{r_1^2 + r_2^2 - 2r_1 r_2 \cos\theta_1}}$. When $r_2 \le b$, l_i becomes $\sqrt{r_2^2 - h^2} - \sqrt{a^2 - h^2}$ and we can simply denote

$$l_i = \min\left(\sqrt{b^2 - h^2} - \sqrt{a^2 - h^2}, \sqrt{r_2^2 - h^2} - \sqrt{a^2 - h^2}\right) \tag{36}$$

for both cases. The possible area that A may locate can be represented by the union of the shadow area S_r (dashed) and the attack area S_a, i.e., $S_r \cup S_a$, and the half of this area can be further denoted as $S_1 = \{(r_1, \theta_1) : a < r_1 \le 1, \cos^{-1}\left(\frac{a}{r_1}\right) + \cos^{-1}\left(\frac{a}{r_2}\right) \le \theta_1 \le \pi\} \cup \{(r_1, \theta_1) : 0 \le r_1 \le a, 0 \le \theta_1 \le \pi\}$. The possible area that B can locate is denoted as

$S_2 = \{(r_2, \theta_2) : a < r_2 \leq 1, 0 \leq \theta_2 \leq 2\pi\}$. The initial load of the green ring can be written as

$$L_i = 2 \iint\limits_{S_2 S_1} l_i ds_1 ds_2, \tag{37}$$

where factor 2 is multiplied to count the another half possible area of A for a given B. Note that here we take only one side of paths because the other side will be counted in the integral when B and A are replaced by each other.

After the attack, when $r_2 > b$, as shown in Supplementary Figure 17(b), the first part of path length inside the ring can be written as $l_f = \sqrt{b^2 - a^2}$. When $r_2 \leq b$, l_f becomes $\sqrt{r_2^2 - a^2}$. We can obtain $l_f = \min\left(\sqrt{b^2 - a^2}, \sqrt{r_2^2 - a^2}\right)$ for both cases. The half of the possible area that A locates can be written as $S_1 = \{(r_1, \theta_1) : a < r_1 \leq 1, \cos^{-1}\left(\frac{a}{r_1}\right) + \cos^{-1}\left(\frac{a}{r_2}\right) \leq \theta_1 \leq \pi\}$. And the possible area that B can locate can be written as $S_2 = \{(r_2, \theta_2) : a < r_2 \leq 1, 0 \leq \theta_2 \leq 2\pi\}$. Then similarly, the load of the ring after the attack can be written as

$$L_f = 2 \iint\limits_{S_2 S_1} l_f ds_1 ds_2, \tag{38}$$

where factor 2 is multiplied to count the another half possible area for A.

Based on the above analysis, the increment of the load on the ring due to the attack can be easily written as

$$\Delta L_{ring} = L_f - L_i, \tag{39}$$

and the load increment of each node with radius b can be written as

$$\Delta L_{node} = \frac{1}{2\pi b} \frac{\partial}{\partial b} \Delta L_{ring}. \tag{40}$$

Modeling the initial load

Similar to the previous theory, in the simplified method, we still divide the way of calculating the initial load of the gray plate with radius b into three cases, including $in - in$ that both ends of the path locate inside the plate, $out - in$ with only one end inside the plate and $out - out$ that both ends locate outside the plate.

For the case of $in - in$, as can be seen in Supplementary Figure 18(a), the path length inside the plate (yellow) can be easily written as $l_{ii} = \sqrt{r_1^2 + r_2^2 - 2r_1 r_2 \cos\theta_1}$. The possible area that A and B locate can be written as $S_1 = \{(r_1, \theta_1) : 0 \leq r_1 \leq b, 0 \leq \theta_1 \leq 2\pi\}$ and

$S_2 = \{(r_2, \theta_2) : 0 \le r_2 \le b, 0 \le \theta_2 \le 2\pi\}$, respectively. Then the initial load from the case of $in - in$ is

$$L_0^{ii} = \frac{1}{2} \iint\limits_{S_2 S_1} l_{ii} ds_1 ds_2, \tag{41}$$

where the factor $\frac{1}{2}$ is to diminish the effect of counting twice.

For the case of $out - in$, as can be seen in Supplementary Figure 18(b), the path length inside the plate (yellow) can be obtained as $l_{oi} = \sqrt{r_1^2 - h^2} + \sqrt{b^2 - h^2}$, where h is the distance from the center to the path and it can be written as $\frac{r_1 r_2 \sin\theta_1}{\sqrt{r_1^2 + r_2^2 - 2r_1 r_2 \cos\theta_1}}$. For A, the possible area $S_1 = \{(r_1, \theta_1) : 0 \le r_1 \le b, 0 \le \theta_1 \le 2\pi\}$. For B, the possible area $S_2 = \{(r_2, \theta_2) : b < r_2 \le 1, 0 \le \theta_2 \le 2\pi\}$. Then the initial load from the case of $out - in$ can be written as

$$L_0^{oi} = \iint\limits_{S_2 S_1} l_{oi} ds_1 ds_2. \tag{42}$$

For the case of $out - out$, as can be seen in Supplementary Figure 18(c), the path length inside the plate (yellow) can be written as $l_{oo} = 2\sqrt{b^2 - h^2}$, where h is the distance from the center to the path and it can be written as $\frac{r_1 r_2 \sin\theta_1}{\sqrt{r_1^2 + r_2^2 - 2r_1 r_2 \cos\theta_1}}$. The half of the possible area that A locates can be written as $S_1 = \{(r_1, \theta_1) : b < r_1 \le 1, \cos^{-1}\frac{b}{r_1} + \cos^{-1}\frac{b}{r_2} \le \theta_1 \le \pi\}$. And the possible area for B can be easily written as $S_2 = \{(r_2, \theta_2) : b < r_2 \le 1, 0 \le \theta_2 \le 2\pi\}$. Then the initial load comes from the case of $out - out$ is

$$L_0^{oo} = \frac{1}{2} \times 2 \iint\limits_{S_2 S_1} l_{oo} ds_1 ds_2, \tag{43}$$

where factor 2 is multiplied to count the another half of the possible area of A, and factor $\frac{1}{2}$ is to diminish the effect of counting twice when A and B are replaced by each other in the integral.

Finally, the initial load of the plate can be written as

$$L_0 = L_0^{ii} + L_0^{oi} + L_0^{oo}, \tag{44}$$

and the load of each node with radius b is

$$L_0^{node} = \frac{1}{2\pi b} \frac{\partial}{\partial b} L_0. \tag{45}$$

Overload analysis

From the above approach, we can get the overload for each node with radius b as $\frac{\Delta L_{node}}{L_0^{node}}$ and for a given tolerance α, we can get the radius of the next-step failure by searching where the condition $\frac{\Delta L_{node}}{L_0^{node}} \leq \alpha$ begins to be satisfied.

Comparison with the previous theory

The results of this simplified theory are also tested and found in excellent agreement with the original theory, as can be seen in Supplementary Figure 19, where the two theories get exactly the same velocity for different tolerances. It is worth noting that because of counting each possible path between two regions, the simplified theory may need heavier computation than the original theory, which only calculates the average path length.

Supplementary References

[1] Delaunay, B. Sur la sphére vide. a la mémoire de georges voronoï. *Bulletin de l'Académie des Sciences de l'URSS. Classe des sciences mathématiques et naturelles* **6**, 793–800 (1934).

[2] Li, F. Real Datasets for Spatial Databases: Road Networks and Points of Interest. `http://www.cs.utah.edu/~lifeifei/SpatialDataset.htm` (2013).

[3] Witthaut, D. & Timme, M. Nonlocal effects and countermeasures in cascading failures. *Phys. Rev. E* **92**, 032809 (2015).

[4] Dobson, I. Obtaining statistics of cascading line outages spreading in an electric transmission network from standard utility data. *Preprint at http://arxiv.org/abs/1507.04277* (2015).
